# Neural Effects of Positive and Negative Incentives during Marijuana Withdrawal

**DOI:** 10.1371/journal.pone.0061470

**Published:** 2013-05-15

**Authors:** Francesca M. Filbey, Joseph Dunlop, Ursula S. Myers

**Affiliations:** 1 Center for Brain Health, School of Behavioral and Brain Science, University of Texas at Dallas, Richardson, Texas, United States of America; 2 Joint Doctoral Program in Clinical Psychology, San Diego State University Department of Psychology/The University of California Department of Psychiatry, San Diego, California, United States of America; University of Granada, Spain

## Abstract

In spite of evidence suggesting two possible mechanisms related to drug-seeking behavior, namely reward-seeking and harm avoidance, much of the addiction literature has focused largely on positive incentivization mechanisms associated with addiction. In this study, we examined the contributing neural mechanisms of avoidance of an aversive state to drug-seeking behavior during marijuana withdrawal. To that end, marijuana users were scanned while performing the monetary incentive delay task in order to assess positive and negative incentive processes. The results showed a group x incentive interaction, such that marijuana users had greater response in areas that underlie reward processes during positive incentives while controls showed greater response in the same areas, but to negative incentives. Furthermore, a negative correlation between withdrawal symptoms and response in the amygdala during negative incentives was found in the marijuana users. These findings suggest that although marijuana users have greater reward sensitivity and less harm avoidance than controls, that attenuated amygdala response, an area that underlies fear and avoidance, was present in marijuana users with greater marijuana withdrawal symptoms. This is concordant with models of drug addiction that involve multiple sources of reinforcement in substance use disorders, and suggests the importance of strategies that focus on respective mechanisms.

## Introduction

Drug-seeking behavior has been attributed to positive reinforcement processes that underlie increased sensitivity to rewarding effects of drugs as well as negative reinforcement processes, such as those that occur during avoidance of aversive effects of withdrawal [1]. In the allostasis model, for example, the development of addiction is characterized by different sources of reinforcement depending on the specific stage of the disorder (i.e., initiation, maintenance, relapse) [2]. For instance, initiation and transition to drug addiction are associated with heightened sensitivity to rewarding stimuli (i.e., positive reinforcement), which is characterized by hyper-responsiveness in the reward neurocircuitry. The allostasis model also describes the later stages of addiction (i.e., maintenance and protracted abstinence) in terms of increased sensitivity to negative stimuli. Later stages are characterized in part by an increased motivation to avoid an aversive state such as continued use of drugs in order to avoid withdrawal symptoms [3] [4], [5], [6]. This later stage, referred to as the ‘dark side’ of drug addiction [3], is associated with the shift towards motivational aspects of withdrawal.

To date, the majority of studies on the neurobiological mechanisms of substance use disorders (SUDs) have focused on the positive reinforcement properties of drugs that lead to drug-seeking behavior. These studies show that areas within the mesocorticolimbic pathway such as the prefrontal cortex (PFC) and ventral striatum (VS) underlie reward craving and motivation for drugs, including marijuana, and their associated cues [7], [8]. Similar findings have also been reported in response to secondary, non-drug (i.e., money) rewards in marijuana users. Using the monetary incentive delay (MID) task to determine reward processes for monetary rewards in marijuana users, Nestor et al. (2010) reported that greater VS response also underlies anticipation of a possible monetary gain. However, sensitivity of the VS to rewards, including non-drug rewards has been inconsistent. In another study utilizing the MID in long-term marijuana users, attenuation of VS response during anticipation of monetary gain was found compared to controls [9]. Discrepancies have been attributed to differences in task design [10].

Unlike the literature on positive reinforcement mechanisms, those associated with negative reinforcement remain relatively unknown. However, the importance of negative reinforcement processes in SUDs is widely supported by animal and human literature illustrating that avoidance of withdrawal symptoms functions as a motivator that, in turn, enhances the incentive value of the drug [11], [12], [13], [14]. Utilizing paradigms such as conditioned place preference, animal studies demonstrate that cues associated with drug withdrawal enhance the incentive value of drug reward cues, and, additionally, are associated with later drug relapse [15] ^,^ [16]. As mentioned earlier, the allostatis theory of addiction suggests that a physiological shift in the experience of drug effects during prolonged use creates a transition from the rewarding effects of the drug to the withdrawal effects of the drug. This theory coincides with behavioral models of addiction that describe continued use of the drug in such chronic states as a desire to avoid negative states such as withdrawal or stress [17], [18]. Human neuroimaging studies have reported that reward-motivation systems (e.g., orbitofrontal cortex, caudate, VS, anterior cingulate) are also involved during negative reinforcement [19] [20]. For instance, using the MID, Kim et al. (2006) reported that the orbitofrontal cortex (OFC) is active during successful avoidance of monetary loss in healthy individuals [21]. Neuroimaging studies have also suggested that reward-motivation systems are differentially affected by drug-seeking behavior, such as withdrawal [22]. For example, smokers in withdrawal showed greater activation in brain regions for incentive salience during smoking cues, which was also found to be positively associated with negative affect [23]. In sum, withdrawal processes involve dysregulation of similar brain regions that underlie acute positive reinforcing effects of the drugs (i.e., reward-motivation circuitry) [24]. The importance of withdrawal in addiction, therefore, suggests its relevance for intervention strategies. In cannabis dependence, however, only recently have withdrawal symptoms been considered a clinically significant syndrome and a potential target for therapies. Indeed, recent studies have shown that 70% of marijuana users relapse due to intense withdrawal symptoms suggesting negative reinforcing effects of marijuana in cannabis dependence [25] [26] [27]. In sum, growing evidence support a marijuana withdrawal syndrome that may drive the high rate of relapse in marijuana dependent individuals.

In this study, we aimed to add to this growing literature by investigating the underlying neural mechanisms that are associated with negative reinforcement processes in marijuana users. To that end, we compared the mechanisms that underlie incentive processes for both positive and negative stimuli in marijuana users during withdrawal and determined the relationship of these mechanisms to withdrawal symptoms. Based on our review of the literature that suggests that avoidance of harm, much like acquisition of reward, is in itself rewarding, we hypothesized that negative incentives will elicit response in similar structures within the reward pathway such as the VS and PFC in marijuana users. We also expected that similar to reward mechanisms, neural mechanisms during avoidance of punishment will be greater in marijuana users compared to non-using controls, and will be positively associated with withdrawal symptoms.

## Materials and Methods

This study was approved by the University of New Mexico and the University of Texas at Dallas Institutional Review Boards. All of the participants provided written informed consent to participate in this study.

### Participants

In this study, 59 heavy marijuana users (MJ) and 27 non-using controls (CON) were recruited from the general population in the Albuquerque metro area (Table 1). Eligibility for the study required right-handedness, having English as the primary language, absence of current or history of psychosis, traumatic brain injury, and MRI contraindications (e.g., pregnancy, non-removable metallic implants, claustrophobia). MJ users were recruited based on self-reported regular marijuana use (i.e., 4 uses per week for at least 6 months) and a positive urinalysis for THC metabolites. Of note, while the criterion was regular use for 6 months preceding participation in the study, for all of those included in the study, the minimum regular marijuana use was 1 year. Based on SCID-I (for DSM-IV) Research Version [28] interviews, 6 participants met criteria for current marijuana abuse and 34 met criteria for current cannabis dependence. Controls were recruited based on self-reported absence of a lifetime history of daily marijuana use and negative urinalysis for THC metabolites. All of the participants were screened via urinalysis for other drugs of abuse and were excluded if drugs (other than marijuana in MJ users) were detected. Based on the timeline followback calendar, the MJ group had a mean duration of marijuana use of 6.2 years (SD = 6.3), mean marijuana use occasions of 3.3 times per day (SD = 1.6) and had a mean marijuana withdrawal checklist (MWC) score of 6.4 (SD = 4.7). Individuals in the control group did not report any marijuana use within the last 9 months. None of the controls met criteria for past cannabis dependence and 3 met criteria for past marijuana abuse. Regarding other drugs of abuse, 12 MJ users were regular tobacco smokers (10 cigarettes per day), one MJ participant reported using crystal meth once a month, eight participants reported using cocaine once a month, one participant reported using cocaine once a week and one person reported using ecstasy once a month. In the CON group, one person reported using ecstasy once a week. No members of CON were current smokers, although six members of CON were former smokers (all tobacco free for at least one year). The members of CON who previously smoked and members of MJ group who smoked (12) did not differ in the number of cigarettes smoked per day at peak use (*t*(60) = .12; *p* = .91). However, among members of CON and MJ groups who drank alcohol, members of the MJ group tended to drink more drinks per occasion (*t*(73) = 1.91; *p* = .061), and more often (*t*(85) = 2.45; *p* = .03, see Table 1).

**Table 1:** Demographic characteristics of marijuana (MJ) and control (CON) groups.

	MJ	CON	
	*Mean (SD)*	*Mean (SD)*	*p*
N	59	27	-
Age	23.49 (6.37)	30.32 (10.09)	* *t*(85) = −3.29; *p*<.001
% Males	78.2%	17.9%	*χ^2^(1) = 30.16; *p*<.001
Years of education	13.28 (2.57)	15.5 (2.41)	* *t*(84) = −3.87; *p*<.001
WASI verbal IQ	54.33 (10.15)	57.22 (9.25)	*t(84) = *−.*213; p = .51*
# Drinking days/90 days	24.03 (24.907)	11 (19.071)	* *t*(85) = 2.45; *p* = .03
Alcohol drinks/Occasion	7.64 (15.33)	1.65 (1.09)	*t*(73) = 1.91; *p* = .06
MID RT	215.67 (25.7)	223.34 (32.6)	*t*(84) = −1.18; *p* = .24
MID accuracy	66.68 (3.77)	66.67 (2.85)	*t*(84) = 0.014; *p* = .99
MID total earnings	43.96 (14.2)	46.71 (12.8)	*t*(83) = −0.848; *p* = .40
Age of initial MJ use	15. 04 (2.63)	−	
Duration of MJ use	8.56 (6.34)	−	
MWC total	6.53 (4.67)	−	
SCID current MJ Dependency	34	−	
# MJ days/90 days	82.52 (12.77)	−	
MCQ total	184 (131)		

* 10 MJ met SCID-I criteria for current alcohol dependence and 5 for current alcohol abuse. None of the controls met criteria for current alcohol abuse or dependence; MCQ = marijuana craving questionnaire; MWC = marijuana withdrawal checklist; WASI = Wechsler Abbreviated Scale of Intelligence; MID = Monetary Incentive Delay task; RT = response time; SCID = Structured Clinical Interview for DSM Disorders.

### Study Procedure

Because our goal was to ascertain the effects of withdrawal during reward processing and studies have shown peak withdrawal symptoms between 2–6 days from day of last use [29], MJ users were asked to abstain from marijuana use for 72-hours prior to the experiment to maximize withdrawal symptoms while minimizing attrition. To verify abstinence, we used a bogus pipeline similar to other reported studies^11^, during which a urinalysis (rather than GC/MS) of THC metabolites was conducted at baseline and also during the experimental session (i.e., following 72-hour abstinence). Although insensitive to 72-hour abstinence, this method has been shown to increase accuracy of self-report [30]. Only those who reported 72-hour abstinence were included in the study. In addition to questionnaires that assessed frequency and quantity of marijuana use, participants also completed the Marijuana Withdrawal Checklist (MWC) [29], [31] to assess current withdrawal symptoms. Following the assessments, they underwent an MRI scan that included a high-resolution structural scan (described below) in addition to a functional MRI scan during cue-reactivity and stop-signal tasks (both not reported here). This was in addition to the MID task to measure positive and negative incentive processes (i.e., anticipation for gains and losses). All participants received monetary compensation for their time and their performance on the MID task.

### fMRI Task

There are many different versions of the MID, but for this study, we utilized the traditional version, which was originally described by Knutson et al. (2000) [32] and previously used in marijuana users^32^. Prior to the experiment, the participants were informed that they would be given their earnings from the MID, which were based on their task performance. Throughout the task, the participants were provided information about their cumulative earnings. Participants received their total earnings in cash at the end of the experiment. The participants were also given explicit instructions that (1) during GAIN trials, they must press the button when the target square is present in order to earn money, (2) during LOSS trials, they must press the button when the target square is present in order to avoid losing money, and (3) during the NEUTRAL trials, their performance will not affect their cumulative earnings, but that their reaction times will be recorded. They were also given a 2-minute practice to ensure understanding of the task. The task consisted of 2 runs of 72×6-second trials (total of 7∶12 minutes) presented in a pseudorandom order. A single trial began with a 250 ms cue screen that informed participants whether the trial was an opportunity to gain money (GAIN), avoid losing money (LOSS) or of no monetary consequence (NEUTRAL). In addition, the cue screen also indicated the amount of money at stake (i.e., $0.20, $1.00 or $5.00). There were 54 GAIN trials, 54 LOSS trials and 36 neutral trials. There were 36 trials for each level of incentive magnitude. In order to be successful during the trial, the participants were instructed to press a button when the target (i.e., gray square) was presented (166–435 ms duration). Success during GAIN trials resulted in gaining the amount of money presented during that trial. Success during LOSS trials resulted in not losing the amount of money presented during the trial. During neutral trials, participants did not gain or lose money, although they were told that their accuracy and response times would be recorded. The target was followed by a delay period between 1165–1934 ms, then by a feedback screen (1650 ms duration) that informed participants whether they were successful or not, in addition to how much money they had gained or lost as well as a running cumulative total of their earnings, which they will receive in cash after the experiment. An adaptive algorithm was implemented to allow a 66% success rate. Because we were interested in incentivization processes, we focused on the anticipation phase of the MID.

### fMRI Acquisition

MRI images were collected using a 3T Siemens whole body scanner with a 12-channel head phased array coil combined with body coil transmission. High resolution structural MRI scans were collected with a multi-echo MPRAGE (MEMPR) sequence with the following parameters: TR/TE/TI = 2300/2.74/900 ms, flip angle = 8°, FOV = 256×256 mm, Slab thickness = 176, Voxel size = 1×1×1 mm, Number of echos = 4, Pixel bandwidth = 650 Hz. fMRI scans were collected using a gradient echo, echoplanar sequence with ramp sampling correction using the intercomissural line (AC-PC) as a reference (TR: 2.0 s, TE: 27 ms, α: 70°, matrix size: 64×64, 32 slices, slice thickness: 3.5 mm, voxel size: 3×3×4 mm^3^). Additionally, in order to improve the signal dropout and warping in the OFC, a tilting acquisition was applied [33]. Images were collected in the oblique axial plane and whole brain coverage was achieved for all participants.

### Data Analyses

T-tests and χ^2^ tests were used to evaluate differences in age, gender, education and alcohol use between MJ and CON groups (Table 1). Continuously scaled self-report variables (i.e., total score on MWC) were examined for skewness and kurtosis to determine the need for normalizing transformations prior to the analyses. Because of differing sample sizes, variances were compared between groups for both percent correct and reaction time.

The fMRI data were pre-processed using slice-time correction and realigned using INRIalign [34] [35]. The entire sample of 86 participants included in these analyses had movement <1 voxel size (an additional 34 were excluded and not reported here due to excessive motion, i.e., >2 mm). Excluded participants did not differ from included participants in gender (χ2 = .983, p = .321), age (*t*(91) = −.970, p = .335, educational attainment (*t*(91) = −.640, p = .548), age of use onset (*t*(89) = 0.10, p = .992), craving symptoms (MCQ; *t*(91) = −1.106, p = .271), or withdrawal symptoms (MWC; *t*(91) = .696, p = .488). Next, using FEAT (fMRI Expert Analysis Tool) Version 5.98, part of FSL (fMRIB’s Software Library) [36], the following pre-statistics processing were performed: non-brain tissue/skull removal using BET (Brain Extraction Tool); spatial smoothing using a Gaussian kernel of FWHM 8 mm^3^; mean-based intensity normalization of all volumes by the same factor; and high-pass temporal filtering (Gaussian-weighted least-squares straight line fitting, with sigma = 50.0 s). Subject-level statistical analyses for each time-series were carried out using FILM (FMRIB's Improved Linear Model) with local autocorrelation correction. Explanatory variables (EVs) were created by convolving the stimulus timing files with a double gamma hemodynamic response function in FEAT. Within each run, the fourteen EVs of interest included anticipation of GAIN for $0.20, $1, and $5, anticipation of LOSS for $0.20, $1, and $5, anticipation of the NEUTRAL condition, acquisition for successful GAIN for $0.20, $1, and $5, acquisition for successful avoidance of LOSS for $0.20, $1, and $5 and acquisition for success in the NEUTRAL condition. Using these EVs, contrasts between the regressors were used to generate contrast maps (i.e., between GAIN anticipation and LOSS anticipation EVs as well as between GAIN vs. NEUTRAL and LOSS vs. NEUTRAL EVs), which were then registered to the high-resolution image using FLIRT (FMRIB’s Linear Image Registration Tool) ^27^ ^28^. Additional contrasts were defined between each level of incentive (i.e., between GAIN $5 anticipation and NEUTRAL anticipation). Group analyses were carried out using FLAME (FMRIB's Local Analysis of Mixed Effects) with group as a fixed factor and subject as random factor [37] [36]. FLAME corrects for unbalanced group sizes by implementing a standard weighted fixed effects model [37]. Statistical maps were registered to the Montreal Neurological Institute (MNI) template with a two-step process (i.e., EPI images to high-resolution image to the 152 brain average MNI template).

The resulting statistical maps were thresholded in order to control for multiple comparisons. We applied cluster-level thresholding as implemented in FEAT where the Z (Gaussianised T/F) statistic images were thresholded using Gaussian Random Field (GRF)-theory-based maximum height thresholding in order to define contiguous clusters [38]. Then each cluster's estimated significance level (from GRF-theory) was compared with the cluster probability threshold [39]. For the main effects of incentive (GAIN, LOSS, NEUTRAL), activity was considered significant if *z*>2.3, with a whole-brain corrected cluster probability of *p*<.05. For large distributed clusters (i.e., >10,000 voxels), the height threshold *z* was increased to 3.3 (i.e., *p*<.001) in order to separate the cluster into smaller, interpretable clusters. For the secondary analyses where effects are expected to be smaller (group-comparisons and correlations), we carried out a two-step statistical thresholding approach. Specifically, a first test at z>2.3 was applied, and where no result meet this threshold, *z*>1.96, with a whole-brain corrected cluster probability of *p*<.05 was applied. Clusters of activation were localized with the Talairach Daemon database implemented in FSL [40] and verified by the Talairach and Tournoux brain atlas.

## Results

The MJ and CON differed in age, gender and education (see Table 1). Thus, all of the between group analyses were co-varied for these variables.

### MID Behavioral Performance

No significant differences in variance for MID percent correct and reaction time were detected between groups via F-test or Levene’s Test. Both groups performed equally well (mean accuracy: 66.63%, mean response time: 219 ms) (Table 1). A partially repeated measures ANOVA for accuracy, with type of incentive (GAIN, LOSS, NEUTRAL) repeated, showed no significant differences during the MID task between groups (*F* (1,83) = 0.05, MSE = 77.7, *p* = 0.83) or incentives (*F* (2,166) = 0.56, MSE = 53.9, *p* = 0.57). A similar partially repeated measures ANOVA for response time showed no significant differences between groups (*F* (1,83) = 1.55, MSE = 4803, *p* = 0.26). A *t*-test found no differences in the total amount won or lost by either group (GAIN: M_CON_ = 39.30, M_MJ_ = 37.96, *t* (83) = −1.2791, *p* = 0.2044; LOSS: M_CON_ = 15.95, M_MJ_ = 15.98, *t* (83) = 0.0335, *p* = 0.97**).**

### Main Effects of Anticipation of Monetary Gains and Losses

In the MJ group, contrasts between the anticipatory periods of a possible gain (GAIN) vs. anticipatory periods of neutral condition (NEUTRAL) showed greater response during GAIN in **5** clusters (Table 2) encompassing the dorsal and ventral striatum (VS), thalamus, inferior frontal gyrus (IFG), orbitofrontal cortex (OFC), and anterior cingulate gyrus (ACG), precentral gyrus, and fusiform gyrus (cluster-corrected *p*<.05, *z* = 3.3). There were no areas of greater activation during NEUTRAL vs. GAIN. Similarly, contrasts between anticipatory periods of possible loss (LOSS) vs. NEUTRAL showed greater response during LOSS in 6 clusters (Table 2) encompassing reward areas including the VS, ventral tegmental area (VTA), insula, OFC, IFG, ACG, and thalamus, in addition to other areas such as the posterior cingulate gyrus and precuneus, (cluster-corrected *p*<.05, *z* = 3.3). In the reverse contrast (i.e., NEUTRAL>LOSS), there was greater activation in the OFC (cluster-corrected *p*<.05, *z* = 2.3). The peak loci of activation are listed in Table 2.

**Table 2:** Peak loci of activation for anticipation of GAIN (vs. NEUTRAL) and LOSS (vs. NEUTRAL).

Cluster	# Voxels	Localization	Z	MNI x, y, z	BA
**1. GAIN anticipation**					
*Marijuana Users (cluster-corrected p<.05, z = 3.3)*					
1	13,187	R Precuneus	5.42	30, −54, 36	−
2	6,359	R Globus Pallidus	6.48	14, 8, −4	−
3	2,021	R Medial Frontal Gyrus	5.12	6, 18, 44	6
4	1,662	R Inferior Frontal Gyrus	5.07	52, 14, 20	9
5	1,433	R Precentral Gyrus	5.42	−46, −8, 46	4
*Controls (cluster-corrected p<.05, z = 2.3)*					
*None*					
*Marijuana Users vs. Controls (cluster-corrected p<.05, z = 1.96)*					
*None*					
**2. LOSS anticipation**					
*Marijuana Users (cluster-corrected p<.05, z = 3.3)*					
1	9,681	L Midbrain/Substantia Nigra	6.72	−6, −28, −14	−
2	3,129	R Medial Frontal Gyrus	5.66	4, 26, 46	8
3	2,069	L Superior Parietal Lobe	5.22	−30, −60, 48	7
4	2,022	R Inferior Parietal Lobe	6.25	38, −48, 44	40
5	401	L Cerebellum	4.8	−20, −84, −14	−
6	380	R Precentral Gyrus	4.82	44, −2, 48	6
*Controls (cluster-corrected p<.05, z = 2.3)*					
*None*					
*Marijuana Users vs Controls (cluster-corrected p<.05, z = 1.96)*					
*None*					

Maximum z-scores, MNI x, y, z co-ordinates and Brodmann areas (BA) are provided for each peak region within the cluster.

Unlike the MJ group, the CON group showed no significant difference during either incentive condition (GAIN, LOSS) when compared to the NEUTRAL condition.

**Figure 1:**
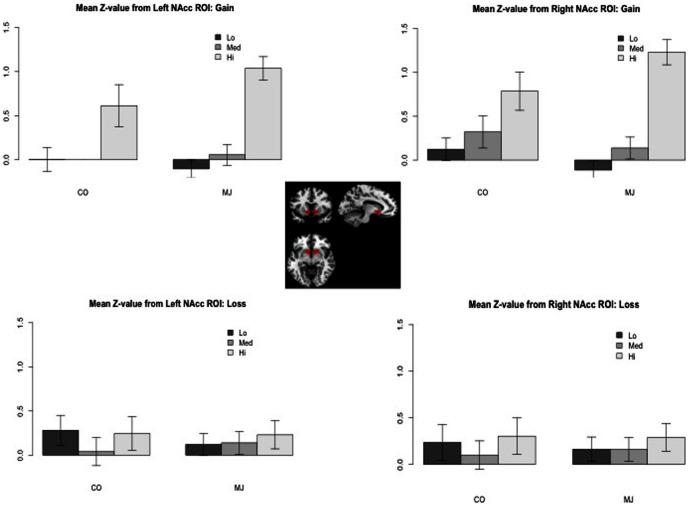
Activation in Nucleus Accumbens during anticipation. Mean *z*-scores per level of magnitude (Lo = $0.20, Med = $1.00, Hi = $5.00) for marijuana users (MJ) and controls (CON) for anticipation of GAIN and LOSS (relative to NEUTRAL) in the nucleus accumbens (NAc). Mean z-scores were extracted using an anatomical mask of the NAc based on the definition described in Filbey et al. (2008).

The data showed that the response of the CON group was sensitive to large magnitudes (i.e., $5.00) for both GAIN and LOSS conditions, but not for the small or medium magnitudes (i.e., $0.20, $1.00) (see bar graphs in Figure 1). Thus, there was attenuation of signal during the incentive conditions when all levels of magnitude were combined. Looking only at high magnitude trials, CON showed greater neural response in several areas during GAIN (vs. NEUTRAL) including VS, OFC, insula, ACG and IFG (cluster-corrected *p*<.05, *z* = 2.3) (see Material S1). High magnitude trials during LOSS (vs. NEUTRAL) in CON also showed greater response in caudate, insula, IFG, OFC and ACG (cluster-corrected *p*<.05, *z* = 2.3). There were no areas of greater activation during NEUTRAL vs. GAIN or NEUTRAL vs. LOSS.

### Differences between MJ and CON

**Figure 2:**
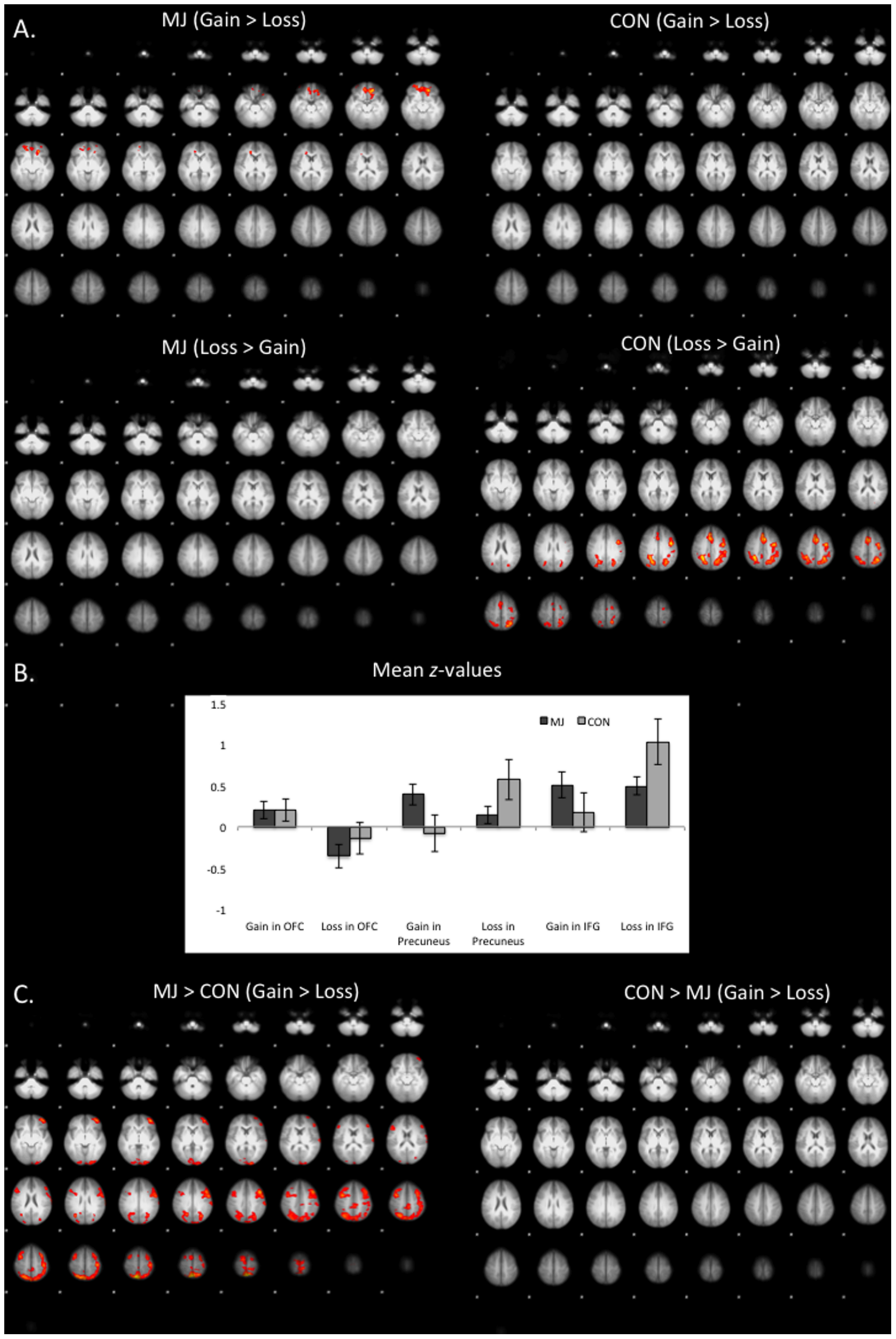
Differences between positive and negative incentivization processes during the anticipation phase. (A) Contrast of GAIN vs. LOSS and LOSS vs. GAIN during the anticipation phase in marijuana users and controls (cluster-corrected *p*<.05, z = 2.3); (B) Mean *z*-scores for marijuana users and controls during GAIN (vs. NEUTRAL) and LOSS (vs. NEUTRAL) in the orbitofrontal cortex (OFC), precuneus, and inferior frontal gyrus (IFG); (C) Contrast of positive and negative incentivization differences (GAIN vs. LOSS) between marijuana users and controls (cluster-corrected *p*<.05, *z* = 1.96). MJ = marijuana group; CON = control group.

Group comparisons were carried out to determine differences in incentive processes between MJ and CON. There was no difference found between groups in the contrasts between incentive conditions (i.e., GAIN, LOSS) vs. NEUTRAL. Between-group differences during GAIN vs. LOSS did not reach the selected height-threshold of z>2.3. However at z>1.96, GAIN vs. LOSS contrasts showed a significant dissociation in neural response to incentive type such that MJ users responded more to GAIN than LOSS in a cluster (size = 16,015 voxels) encompassing several areas including OFC and cingulate gyrus (cluster-corrected *p*<.05, *z* = 1.96), whereas CON had greater BOLD response to LOSS compared to GAIN in OFC (cluster-corrected *p*<.05, *z* = 1.96) (Figure 2) (peaks are listed in Table 3). The double subtraction of MJ_(GAIN>LOSS)_>CON_(GAIN>LOSS)_ was greater in MJ_(GAIN>LOSS)_ compared to CON_(GAIN>LOSS)_ in a cluster with peaks in the precuneus, middle frontal gyrus, post-central gyrus and parietal lobe (cluster-corrected *p*<.05, *z* = 1.96) (Figure 2C, Table 3).

**Table 3:** Peak loci of activation differences for anticipation of LOSS vs. GAIN and_GAIN vs. LOSS.

Cluster	# Voxels	Localization	Z	MNI x, y, z	BA
**1. LOSS vs. GAIN anticipation**					
*Marijuana Users (cluster-corrected p<.05, z = 2.3)*					
*None*					
*Controls (cluster-corrected p<.05, z = 2.3)*					
1	4,200	L Inferior Frontal Gyrus	3.78	−42, 2, 36	6
2	2,457	R Supramarginal gyrus	3.95	30, −46, 40	7
3	1,378	L Medial Frontal Gyrus	3.79	−4, 14, 46	24
*Marijuana Users>Controls (cluster*					
*None*					
**4. GAIN vs. LOSS anticipation**					
*Marijuana Users (cluster-corrected p<.05, z = 2.3)*					
1	1,576	L Frontal Pole/Orbitofrontal Cortex	3.83	−18, 40, −16	−
1		L Anterior Cingulate/Orbitofrontal Cortex	3.5	−12, 30, −14	10
1		L Medial Frontal Gyrus	3.4	−12, 34, −14	10
1		R Frontal Pole	3.23	22, 46, −10	11
1		L Orbitofrontal gyrus	3.16	−8, 40, −16	10
*Controls (cluster-corrected p<.05, z = 2.3)*					
*None*					
*Marijuana Users>Controls (cluster-corrected p<.05, z = 1.96)*					
1	16,015	R Precuneus	4.05	8, −66, 66	7
1		L Precuneus	3.77	−2, −60, 70	7
1		L Middle Frontal Gyrus	3.42	−44, 16, 42	6
1		L Postcentral Gyrus	3.32	−42, −24, 58	1
1		L Middle Frontal Gyrus	3.32	−38, 52, 0	11

Maximum z-scores, MNI x, y, z co-ordinates and Brodmann areas (BA) are provided for each peak region within the cluster.

### Relationship of Marijuana Behavioral Symptoms and Neural Response to Incentives

**Figure 3:**
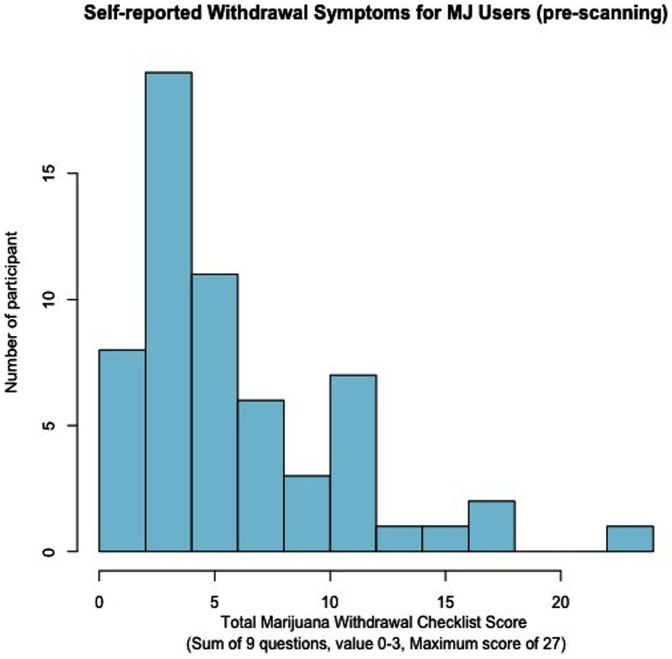
Distribution of Total Marijuana Checklist Scores. The possible range of Marijuana Withdrawal Checklist (MWC) total score is 0–27, which are derived from 9 items scored using a 4-point Likert scale (0 = none, 1 = mild, 2 = moderate and 3 = severe).

**Figure 4:**
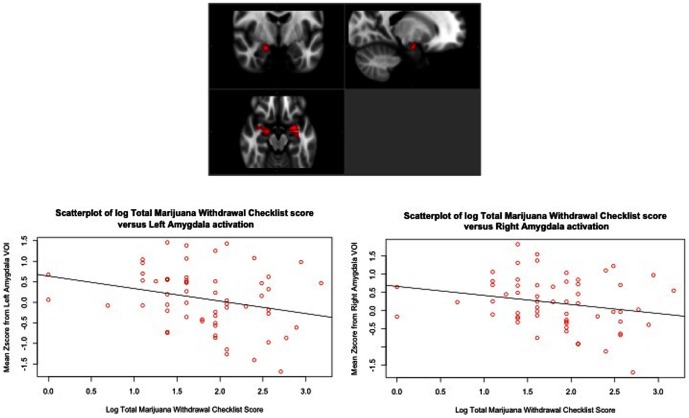
Negative correlations between marijuana withdrawal checklist (MWC) and anticipation of LOSS (vs. NEUTRAL) BOLD response in bilateral amygdala (shown below). Significant at cluster-corrected *p*<.05, *z* = 1.96. For the scatterplots, an anatomical mask of the amygdala was created based on the definition described by Tzourio-Mazoyer and colleagues [64].

In order to determine how these incentive processes relate to the behavioral symptoms of marijuana abuse and dependence, we performed a correlation analysis between the total scores from the MWC and the statistical maps for (1) LOSS (vs. NEUTRAL) and (2) GAIN (vs. NEUTRAL). Because there was a non-Gaussian distribution of the total MWC scores (Figure 3), we performed a log-transformation of the total MWC scores for these analyses. These correlations did not reach the height threshold of z = 2.3; however, they showed a significant negative association between MWC scores and neural response during GAIN (vs. NEUTRAL) in a cluster (size = 2,774 voxels) with peaks in the OFC and ACG at cluster-corrected *p*<.05, *z* = 1.96. Similarly, we found a negative correlation during LOSS (vs. NEUTRAL) in a cluster (size = 44,360 voxels) encompassing areas involved in reward processes such as the OFC and striatum (cluster-corrected *p*<.05, *z* = 1.96). In addition, there was also a negative correlation in areas within the fear/avoidance network, which included the amygdala and hippocampus (Figure 4) (peaks are listed in Table 4).

**Table 4:** Peak loci of activation during correlation between total marijuana withdrawal checklist (MWC) scores and BOLD response during the anticipation phase.

Cluster	Localization	Z	MNI x, y, z	BA
**1. GAIN anticipation** *(cluster-corrected p<.05, z = 1.96),* 1 cluster (size = 2,774 voxels) *negative correlation				
1	R Frontal Pole/Orbitofrontal Cortex	3.46	42, 60, −18	10
1	R Cingulate Gyrus/Supplementary Motor Cortex	3.2	14, 4, 42	24
1	R Anterior Cingulate Gyrus	3.18	16, 40, 4	32
1	R Frontal Pole/Orbitofrontal Cortex	3.08	44, 56, −8	10
1	R Anterior Cingulate Gyrus	2.99	12, 48, −10	32
**2. LOSS anticipation** *(cluster-corrected p<.05, z = 1.96)*, 1 cluster (size = 44,360 voxels) *negative correlation				
1	R Fusiform Gyrus	4.49	2, −82, −26	−
1	R Postcentral Gyrus	4.22	36, −36, 62	40
1	R Putamen	4.09	28, −20, −2	−
1	R Fusiform Gyrus	3.96	46, −44, −18	37
1	L Cerebellum	3.92	−2, −48, −28	−
1	R Postcentral Gyrus	3.8	36, −26, 58	4
1	L Parahippocampus/Hippocampus	3.63	−22, −34, 0	−
1	R Parahippocampus/Hippocampus	3.52	38, −28, −2	−
1	L Amygdala	3.17	−28, −8, −6	−

Maximum z-scores, MNI x, y, z co-ordinates and Brodmann areas (BA) are provided for each peak region within the cluster.

We also analyzed the associations between the BOLD response to GAIN and LOSS with SCID symptoms for current marijuana abuse and dependence. We found no correlations (positive or negative) between BOLD response and symptom count for marijuana abuse or dependence (cluster-corrected *p*<.05, *z* = 1.96).

### Manipulation Check

Similar to other studies that compare substance abusing to non-abusing groups, we found differences in substance use other than the primary drug of abuse (i.e., marijuana). Specifically, the two groups differed in frequency of alcohol use. To disentangle the confounding effects of alcohol use, we carried out an additional analysis on a sub-group of the sample with matched frequency of alcohol use. We matched 25 MJ users’ mean drinks per drinking day with that of 25 controls [Mean (SD): MJ = 1.8 (1.42), CON = 1.38 (1.42)]. Of note, we only selected MJ users who also did not have other drug use thereby creating a test of frequency of alcohol use-matched MJ-only users and controls. Contrasts of these two smaller groups showed that the MJ group had greater activation in OFC and inferior frontal gyrus/anterior insula region at height threshold of z = 1.96 (cluster-corrected *p*<.05). There were no regions where CON had greater activation compared to MJ. This suggests that despite smaller power to detect differences due to the smaller sample size, the observed effects remained after removing the confounding effects of alcohol and other drug use. Thus, the differences between the MJ and CON in BOLD response to GAIN and LOSS were not due to differences in age, gender, education, or alcohol/other substance use.

Additionally, we tested whether those who are more severe marijuana users may be driving the difference between the groups. To that end, we compared the 34 MJ users who met SCID diagnosis of cannabis dependence to the CON group. This analysis revealed no significant group difference (cluster-threshold *p*<.05, *z* = 2.3), suggesting the possibility that the effect is driven by the non-dependent marijuana users.

With regards to the correlation between MWC and LOSS>NEUTRAL, we examined factors that may influence this effect such as age of onset and duration of use. Adding these two past MJ use variables to our model independently showed that the activations (that include amygdala) remain at height threshold of z = 1.96 (cluster-corrected *p*<.05). Similarly, because of the relevance of craving to withdrawal symptoms, we included craving scores (based on Marijuana Craving Questionnaire) to this model, and, as suspected, craving was also correlated with LOSS>NEUTRAL contrast, but only for the high-magnitude trials (i.e., $5) in the R lingual gyrus (16,994 voxels, max *z* = 4.15) and R middle frontal gyrus (2,440 voxels, max *z* = 3.82) (cluster-corrected *p*<.05, *z* = 1.96). This result did not survive z = 2.3 height threshold.

To further explore our findings of greater activation in reward areas during anticipation of punishment, we determined whether this activation was sustained during the outcome phase of the task (i.e., feedback of successful gain or avoidance of loss). This analysis showed that in the MJ group, there was significant activation in the lingual gyrus and anterior cingulate during GAIN outcome (cluster-corrected *p*<.05, *z* = 3.3) and in the inferior frontal gyrus and inferior parietal lobe in the LOSS outcome (cluster-corrected *p*<.05, *z* = 2.3). In the CON group, there was greater response in the lingual gyrus, precuneus, inferior parietal lobe, anterior cingulate gyrus, parahippocampal gyrus, superior temporal gyrus, middle frontal gyrus, inferior frontal gyrus, caudate and globus pallidus during GAIN outcome (cluster-corrected *p*<.05, *z* = 3.3) and in the inferior frontal gyrus and cerebellum in the LOSS outcome (cluster-corrected *p*<.05, *z* = 2.3). Contrasts of the groups showed no significant difference between the groups during GAIN outcome or LOSS outcome (cluster-corrected *p*<.05, *z* = 2.3). Peaks are listed in Table 5.

**Table 5:** Peak loci of activation during the outcome phase for GAIN (vs. NEUTRAL), LOSS (vs. NEUTRAL).

Cluster	# voxels	Localization	Z	x, y, z	BA	
**1. GAIN outcome**						
*Marijuana Users (cluster-corrected p<.05, z = 3.3)*						
1	10,034	R Lingual Gyrus	5.62	22, −88, −8	18	
2	2,161	Anterior Cingulate Gyrus	4.99	0, 44, 4	32	
*Controls (cluster-corrected p<.05, z = 3.3)*						
1	11,156	R Lingual Gyrus	5.06	28, −82, −8	18	
2	1,489	L Precuneus	4.43	−30, −44, 46	7	
3	1,376	R Inferior Parietal Lobe	4.53	46, −34, 40	40	
4	511	Anterior Cingulate Gyrus	4.5	0, 42, −4	−	
5	493	L Parahippocampal Gyrus	4.13	−24, 2, −14	34	
6	365	R Superior Temporal Gyrus	4.04	62, −14, 2	22	
7	359	R Cerebellum	4.5	20, −68, −50	−	
8	302	R Caudate Nucleus	4.26	12, 10, −8	−	
9	288	R Middle Frontal Gyrus	4.52	34, 56, −8	10	
10	274	L Inferior Frontal Gyrus	3.91	−44, 42, −2	46	
11	229	R Globus Pallidus	3.9	26, −12, 2	−	
12	210	R Cingulate Gyrus	4.03	4, −32, 28	23	
**2. LOSS outcome**						
*Marijuana Users (cluster-corrected p<.05, z = 2.3)*						
1	4,944	L Inferior Frontal Gyrus	5.17	−40, 48, −10		10
2	1,708	L Cerebellum	4.17	−36, −78, −38		−
3	1,504	L Inferior Parietal Lobe	4.15	−46, −50, 44		40
*Controls (cluster-corrected p<.05, z = 2.3)*						
1	15,115	L Inferior Frontal Gyrus	4.14	−42, 38, 4	46	
2	6,998	L Cerebellum	3.82	−6, −72, −30	−	

Maximum z-scores, MNI x, y, z co-ordinates and Brodmann areas (BA) are provided for each peak region within the cluster.

## Discussion

Despite similar behavioral task performance during anticipation of monetary gains and losses between marijuana users and non-using controls, our findings suggest a difference between type of incentive and group, such that marijuana users have greater neural response to positive incentives (relative to negative incentives), while non-using controls have greater neural response to negative incentives (relative to positive incentives). Although no statistically significant group difference emerged, the level of VS response was indeed greater during positive incentives in marijuana users that support findings by Nestor et al. [41]. Increased response in VS during anticipation of monetary rewards has been inconsistent [42], [43], and discrepancies between findings could be attributed to differences in populations, task parameters, and/or other factors such as risk-taking bias [44], expectancies [45], as well as maturational differences [46].

In the current study, we aimed to determine processes related to the valuation of negative incentives (i.e., anticipation of loss) as it relates to marijuana use. Our findings showed that negative incentives elicited response from similar regions underlying reward processes in MJ users, which is consistent with findings in alcohol dependent individuals [43]. These findings are in accord with both animal and human studies that suggest a role of the VS in processing aversive stimuli through processes involved in learning, prediction errors and decision-making [47]. The primary role of the VS has been associated with general anticipation and motivation for rewarding stimuli [48], [49] [50]. For instance, studies have described activation in the VS as being predictive of relative incentive motivation suggesting its involvement in reward valuation [51]. Others have implicated its involvement in sensorimotor functions during incentive motivation as obstruction of the VS disrupts active reward-seeking and harm avoidance behavior in animals [52]. Taken together, VS modulates behavior/response to any type of salient stimuli, whether positive/rewarding or negative/aversive, and, therefore, plays a broad role in incentive motivational processes.

Our findings also demonstrated differences between group and type of incentive such that marijuana users have greater neural response to positive incentives and less neural response to negative incentives than controls. This suggests that marijuana users, unlike non-users, are more sensitive to positive incentives, and less sensitive to negative incentives. This latter point is concordant with a study by Wrase and colleagues (2007) that showed a similar pattern of decreased response during LOSS, albeit at trend-level significance, where alcohol dependent individuals had less activation in the VS compared to controls during LOSS [43]. Of note, the controls’ neural response to reward only reached significance in the high-response condition (i.e., $5.00). Thus, we cannot rule out the idea that the difference may be driven by the blunted response from the controls. Interestingly, the MJ users did not differ from controls in the outcome phase of the task for either positive or negative rewards highlighting the greater influence of anticipatory processes in drug-seeking behavior.

We also determined the behavioral associations of this finding. Our analyses yielded an unexpected finding of negative association between withdrawal symptoms and neural response during anticipation of loss in several brain networks. Specifically, we found that the greater the number of withdrawal symptoms, the less the response not only in areas that underlie reward processes, but also in areas that are involved in fear/avoidance processes such as the amygdala. A similar pattern of negative associations in the amygdala was also found with craving, albeit to only the high magnitude trials. This is not surprising given the relatedness of craving to the withdrawal syndrome. Indeed, disrupted amygdala activation has been suggested to underlie pathological avoidance behavior. In healthy adults, greater BOLD response in the amygdala was associated with cues for LOSS. The authors posited that the response magnitude of the amygdala to possible losses may be a mechanism for maladaptive avoidance behavior in SUDs [53]. Additionally, given that compared to the controls, the marijuana users showed attenuated response in these areas, these negative correlations suggest that greater aberration in neural response (i.e., less activation) during the valuation of negative incentives, the more likely they are to experience greater problems with marijuana (i.e., more withdrawal symptoms), which likely contribute to higher risk for continued use and/or relapse in these individuals. Our findings of a lack of correlation between BOLD response to GAIN and to LOSS with marijuana abuse and dependence symptoms, and, past marijuana use measures such as age of onset of use or duration suggests that these findings are specific to withdrawal symptoms rather than to the general concept of dependence.

The amygdala is postulated to underlie fear conditioning [54], encoding fear memories [55], signaling negative outcomes [56], and extinction of conditioned fear [57]. Our findings of attenuated amygdala activation in MJ users suggest deficiencies in learning and encoding negative stimuli. Recent studies also suggest that areas within the reward network such as the striatum functionally modulate the amygdala. Thus, these results suggest that signal from the amygdala may be attenuated such that individuals with high withdrawal do not avoid harmful stimuli, which is in accordance with an approach bias in substance abusers.

The double dissociation between users and non-users and incentivization processes (i.e., positive, negative) strongly support the theory of aberrant approach-bias in marijuana users. Our findings indicate that the brains of marijuana users are more strongly responsive to positive incentive motivation whereas non-using controls are more strongly responsive to negative incentive motivation. As important, these effects are not evident in the outcome or receipt phase whereby both groups showed greater response to the receipt of reward but not to the successful avoidance of loss, and, there were no significant difference between groups. These findings are in accord with the growing literature on the disruption in regulatory and motivational processes in heavy marijuana users. For example, dose-related, persistent monetary-based decision-making impairments (using Iowa Gambling Task, IGT) that illustrate enhanced motivation for immediate rewards (i.e., getting high) despite negative consequences have been reported in abstinent marijuana users (i.e. 28 days) [58]. These deficits in decision-making have paralleled neural alterations such as greater activation in the ventromedial PFC (vmPFC), an important area for reward-based decision making [59]. Interestingly, others have also reported that activation during decision-making tasks, particularly during anticipation of loss predicted change in marijuana use at 6-month follow-up [60]. In an interesting study that aimed to determine the approach vs. avoidance bias in marijuana users, they found no difference between approach-bias neural activations between MJ users and controls. They did find, however, that within the MJ using group, there was a positive association between total lifetime marijuana use and approach-bias activation in PFC and limbic areas. Further, approach-bias activations in the dorsolateral prefrontal cortex (PFC) and anterior cingulate gyrus (ACG) predicted severity of marijuana use at 6-month follow-up. The authors suggest that the difference between approach and avoidance bias responses in the DLPFC and ACG may identify individuals at risk for cannabis dependence [61]. In sum, our findings along with the existing literature on cannabis dependence provide strong evidence for a pathological approach-bias, which presents as deficits in reward-motivation, decision-making processes. All of which likely contribute to susceptibility towards addictive behavior and hinder treatment success.

In summary, our findings suggest that current marijuana users experience sensitivity to salient stimuli whether positive or negative. This finding supports the somatic marker theory of addiction, which posits that an overactive mesolimbic reward/approach circuitry and a deficient frontocortical fear/avoidance system is the underlying mechanism that contributes to impulsivity and/or poor decision-making that leads to SUDs [62], [63]. Indeed, our findings support this theory by demonstrating hypersensitivity in the reward circuitry, and attenuation in areas in the fear/avoidance network, especially in those with greater withdrawal symptoms. These oppositional forces may then work together to drive continued drug use and elevate risk for relapse. These findings provide neurobiological evidence for a dissociation of positive and negative incentive processes in marijuana abuse and dependence, and suggest that compared to non-users, marijuana users are more sensitive to positive reinforcement. This is in line with clinical reports of dissociation in personality measures (i.e., novelty seeking and harm avoidance) as they relate to treatment success [11].

### Limitations and Conclusions

Interpretation of these findings should consider that abstinence from marijuana was based on self-report, and not confirmed by THC quantification. Variability in actual length of abstinence could partially account for the level of withdrawal symptoms and, therefore, fMRI response. However, because self-reported date of last use across all of the participants was the day of their baseline session (i.e., maximum length of abstinence = 3 days prior to their fMRI scan), actual variability may be minimal. Caution should also be taken given the difference across the groups in age, gender and alcohol use. While we controlled for these differences statistically, remaining effects may still be contributing to the differences described. It is also important to keep in mind that an ideal experiment of withdrawal effects would be a pre-post withdrawal design. We are limited in how much we can interpret these cross-sectional findings, as we do not know precisely the reward sensitivity of the non-withdrawn MJ users. Of note, however, to answer the question of whether withdrawal symptoms alone rather than earlier age of onset or longer duration of use contributed to this effect, we also co-varied for age of onset and duration of use. These analyses showed that the activation that included the amygdala remained (cluster-corrected *p*<.05, *z* = 1.96). Future fMRI studies that focus on NAc should also consider a smaller smoothing kernel (∼4 mm) given the relatively small size of this structure. Because we were interested in both cortical and sub-cortical areas, we applied an 8 mm smoothing kernel on the whole brain, therefore, inferences with regards to NAc activation should consider this limitation. Lastly, we utilized the originally designed version of the MID task with fixed intervals, which may increase susceptibility of the results to low frequency noise. Interpretation of the present findings must take this into account as the potential low frequency noise could have minimized the effects.

To conclude, the neural abnormalities underlying negative reinforcement may be the pathophysiology that underlies both difficulties in protracted abstinence and inter-individual variability in risk for withdrawal and relapse particularly in the earlier stages of addiction (i.e., non-dependent users). Our findings suggest that consideration of positive and negative incentive processes is important for the future development of more effective treatment strategies that might emphasize one process over another. Specifically, identification of these mechanisms could lead to a better understanding of the cognitive processes and biological systems involved in the various stages of marijuana abuse and inform treatment options that could alleviate withdrawal symptoms.

## Supporting Information

Material S1 **Effects of incentive magnitude.**
(DOCX)

## References

[1] Jarvis MJ (1994) A profile of tobacco smoking. Addiction 89: 1371–1376. 7841845 10.1111/j.1360-0443.1994.tb03732.x

[2] Koob GF, Le Moal M (2001) Drug addiction, dysregulation of reward, and allostasis. Neuropsychopharmacology : official publication of the American College of Neuropsychopharmacology 24: 97–129. 11120394 10.1016/S0893-133X(00)00195-0

[3] Koob GF, Le Moal M (2005) Plasticity of reward neurocircuitry and the 'dark side' of drug addiction. Nature neuroscience 8: 1442–1444. 16251985 10.1038/nn1105-1442

[4] Koob GF, Le Moal M (2008) Addiction and the brain antireward system. Annual review of psychology 59: 29–53. 10.1146/annurev.psych.59.103006.093548 18154498

[5] Koob GF, Le Moal M (1997) Drug abuse: hedonic homeostatic dysregulation. Science 278: 52–58. 9311926 10.1126/science.278.5335.52

[6] Roberts AJ, Heyser CJ, Cole M, Griffin P, Koob GF (2000) Excessive ethanol drinking following a history of dependence: animal model of allostasis. Neuropsychopharmacology : official publication of the American College of Neuropsychopharmacology 22: 581–594. 10788758 10.1016/S0893-133X(99)00167-0

[7] Filbey FM, Claus E, Audette AR, Niculescu M, Banich MT, et al (2008) Exposure to the taste of alcohol elicits activation of the mesocorticolimbic neurocircuitry. Neuropsychopharmacology 33: 1391–1401. 17653109 10.1038/sj.npp.1301513 PMC2856647

[8] Filbey FM, Schacht JP, Myers US, Chavez RS, Hutchison KE (2009) Marijuana craving in the brain. Proceedings of the National Academy of Sciences of the United States of America 106: 13016–13021. 19651613 10.1073/pnas.0903863106 PMC2716383

[9] van Hell HH, Vink M, Ossewaarde L, Jager G, Kahn RS, et al (2010) Chronic effects of cannabis use on the human reward system: An fMRI study. European Neuropsychopharmacology 20: 153–163. 20061126 10.1016/j.euroneuro.2009.11.010

[10] Hommer DW, Bjork JM, Gilman JM (2011) Imaging brain response to reward in addictive disorders. Annals of the New York Academy of Sciences 1216: 50–61. 21272010 10.1111/j.1749-6632.2010.05898.x

[11] Leventhal AM, Waters AJ, Boyd S, Moolchan ET, Heishman SJ, et al (2007) Associations between Cloninger's temperament dimensions and acute tobacco withdrawal. Addict Behav 32: 2976–2989. 17624682 10.1016/j.addbeh.2007.06.014 PMC2080877

[12] Pomerleau OF, Fagerstrom KO, Marks JL, Tate JC, Pomerleau CS (2003) Development and validation of a self-rating scale for positive- and negative-reinforcement smoking: The Michigan Nicotine Reinforcement Questionnaire. Nicotine Tob Res 5: 711–718. 14577987 10.1080/1462220031000158627

[13] Hutcheson DM, Everitt BJ, Robbins TW, Dickinson A (2001) The role of withdrawal in heroin addiction: enhances reward or promotes avoidance? Nat Neurosci 4: 943–947. 11528427 10.1038/nn0901-943

[14] Cherek DR, Bennett RH, Kelly TH, Steinberg JL, Benowitz NL (1989) Effects of nicotine gum and tobacco smoking on human avoidance responding. Pharmacology, biochemistry, and behavior 32: 677–681. 10.1016/0091-3057(89)90017-8 2740422

[15] Scott D, Hiroi N (2010) Emergence of dormant conditioned incentive approach by conditioned withdrawal in nicotine addiction. Biological psychiatry 68: 726–732. 20598291 10.1016/j.biopsych.2010.05.017 PMC2949488

[16] Sanchis-Segura C, Spanagel R (2006) Behavioural assessment of drug reinforcement and addictive features in rodents: an overview. Addiction biology 11: 2–38. 16759333 10.1111/j.1369-1600.2006.00012.x

[17] Baker TB, Piper ME, McCarthy DE, Majeskie MR, Fiore MC (2004) Addiction motivation reformulated: an affective processing model of negative reinforcement. Psychological review 111: 33–51. 14756584 10.1037/0033-295X.111.1.33

[18] Eissenberg T (2004) Measuring the emergence of tobacco dependence: the contribution of negative reinforcement models. Addiction 99 Suppl 15–29. 10.1111/j.1360-0443.2004.00735.x 15128378

[19] Gottfried JA, O'Doherty J, Dolan RJ (2003) Encoding predictive reward value in human amygdala and orbitofrontal cortex. Science 301: 1104–1107. 12934011 10.1126/science.1087919

[20] Baxter MG, Murray EA (2002) The amygdala and reward. Nat Rev Neurosci 3: 563–573. 12094212 10.1038/nrn875

[21] Kim H, Shimojo S, O'Doherty JP (2006) Is avoiding an aversive outcome rewarding? Neural substrates of avoidance learning in the human brain. PLoS biology 4: e233. 16802856 10.1371/journal.pbio.0040233 PMC1484497

[22] Gloria R, Angelos L, Schaefer HS, Davis JM, Majeskie M, et al (2009) An fMRI investigation of the impact of withdrawal on regional brain activity during nicotine anticipation. Psychophysiology 46: 681–693. 19490513 10.1111/j.1469-8986.2009.00823.x PMC2706918

[23] McClernon FJ, Kozink RV, Rose JE (2008) Individual differences in nicotine dependence, withdrawal symptoms, and sex predict transient fMRI-BOLD responses to smoking cues. Neuropsychopharmacology : official publication of the American College of Neuropsychopharmacology 33: 2148–2157. 17987060 10.1038/sj.npp.1301618

[24] Weiss F, Koob GF (2001) Drug addiction: functional neurotoxicity of the brain reward systems. Neurotoxicity research 3: 145–156. 15111266 10.1007/BF03033235

[25] Levin KH, Copersino ML, Heishman SJ, Liu F, Kelly DL, et al (2010) Cannabis withdrawal symptoms in non-treatment-seeking adult cannabis smokers. Drug and alcohol dependence 111: 120–127. 20510550 10.1016/j.drugalcdep.2010.04.010 PMC2930056

[26] Copersino ML, Boyd SJ, Tashkin DP, Huestis MA, Heishman SJ, et al (2006) Cannabis withdrawal among non-treatment-seeking adult cannabis users. The American journal on addictions/American Academy of Psychiatrists in Alcoholism and Addictions 15: 8–14. 10.1080/10550490500418997 16449088

[27] Cooper ZD, Haney M (2008) Cannabis reinforcement and dependence: role of the cannabinoid CB1 receptor. Addiction biology 13: 188–195. 18279497 10.1111/j.1369-1600.2007.00095.x PMC2731704

[28] First MB, Spitzer RL, Miriam G, Williams JBW (2002) Structured Clinical Interview for DSM-IV-TR Axis I Disorders, Research Version, Non-patient Edition. In: Institute NYSP, editor. New York: Biometrics Research.

[29] Budney AJ, Moore BA, Vandrey RG, Hughes JR (2003) The time course and significance of cannabis withdrawal. J Abnorm Psychol 112: 393–402. 12943018 10.1037/0021-843x.112.3.393

[30] Roese N, Jamieson D (1993) Twenty years of bogus pipeline research: A critical review and meta-analysis. Psychological bulletin 114: 363–375.

[31] Budney AJ, Moore BA (2002) Development and consequences of cannabis dependence. Journal of clinical pharmacology 42: 28S–33S. 12412833 10.1002/j.1552-4604.2002.tb06000.x

[32] Knutson B, Westdorp A, Kaiser E, Hommer D (2000) FMRI visualization of brain activity during a monetary incentive delay task. NeuroImage 12: 20–27. 10875899 10.1006/nimg.2000.0593

[33] Deichmann R, Gottfried JA, Hutton C, Turner R (2003) Optimized EPI for fMRI studies of the orbitofrontal cortex. NeuroImage 19: 430–441. 12814592 10.1016/s1053-8119(03)00073-9

[34] Freire L, Mangin JF (2001) Motion correction algorithms may create spurious brain activations in the absence of subject motion. Neuroimage 14: 709–722. 11506543 10.1006/nimg.2001.0869

[35] Freire L, Roche A, Mangin JF (2002) What is the best similarity measure for motion correction in fMRI time series? IEEE Transactions on Medical Imaging 21: 470–484. 12071618 10.1109/TMI.2002.1009383

[36] Smith SM, Jenkinson M, Woolrich MW, Beckmann CF, Behrens TE, et al (2004) Advances in functional and structural MR image analysis and implementation as FSL. NeuroImage 23 Suppl 1S208–219. 15501092 10.1016/j.neuroimage.2004.07.051

[37] Beckmann CF, Jenkinson M, Smith SM (2003) General multilevel linear modeling for group analysis in FMRI. Neuroimage 20: 1052–1063. 14568475 10.1016/S1053-8119(03)00435-X

[38] Worsley K, Marrett S, Neelin P, Vandal A, Friston K, et al (1996) A unified statistical approach for determining significant signals in images of cerebral activation. Hum Brain Mapp 4: 58–73. 20408186 10.1002/(SICI)1097-0193(1996)4:1<58::AID-HBM4>3.0.CO;2-O

[39] Worsley KJ (2001) Statistical analysis of activation images. Functional MRI: An Introduction to Methods: Ch. 14.

[40] Lancaster JL, Woldorff MG, Parsons LM, Liotti M, Freitas CS, et al (2000) Automated Talairach atlas labels for functional brain mapping. Human Brain Mapping 10: 120–131. 10912591 10.1002/1097-0193(200007)10:3<120::AID-HBM30>3.0.CO;2-8 PMC6871915

[41] Nestor L, Hester R, Garavan H (2010) Increased ventral striatal BOLD activity during non-drug reward anticipation in cannabis users. NeuroImage 49: 1133–1143. 19631753 10.1016/j.neuroimage.2009.07.022 PMC2764826

[42] Beck A, Schlagenhauf F, Wustenberg T, Hein J, Kienast T, et al (2009) Ventral striatal activation during reward anticipation correlates with impulsivity in alcoholics. Biol Psychiatry 66: 734–742. 19560123 10.1016/j.biopsych.2009.04.035

[43] Wrase J, Schlagenhauf F, Kienast T, Wustenberg T, Bermpohl F, et al (2007) Dysfunction of reward processing correlates with alcohol craving in detoxified alcoholics. NeuroImage 35: 787–794. 17291784 10.1016/j.neuroimage.2006.11.043

[44] Schneider S, Peters J, Bromberg U, Brassen S, Miedl SF, et al (2012) Risk taking and the adolescent reward system: a potential common link to substance abuse. Am J Psychiatry 169: 39–46. 21955931 10.1176/appi.ajp.2011.11030489

[45] Wilson SJ, Sayette MA, Delgado MR, Fiez JA (2008) Effect of smoking opportunity on responses to monetary gain and loss in the caudate nucleus. Journal of abnormal psychology 117: 428–434. 18489219 10.1037/0021-843X.117.2.428 PMC2626251

[46] Bjork JM, Smith AR, Chen G, Hommer DW (2010) Adolescents, adults and rewards: comparing motivational neurocircuitry recruitment using fMRI. PloS one 5: e11440. 20625430 10.1371/journal.pone.0011440 PMC2897849

[47] Delgado MR, Li J, Schiller D, Phelps EA (2008) The role of the striatum in aversive learning and aversive prediction errors. Philosophical transactions of the Royal Society of London Series B, Biological sciences 363: 3787–3800. 18829426 10.1098/rstb.2008.0161 PMC2607367

[48] Cooper JC, Hollon NG, Wimmer GE, Knutson B (2009) Available alternative incentives modulate anticipatory nucleus accumbens activation. Social cognitive and affective neuroscience 4: 409–416. 19843618 10.1093/scan/nsp031 PMC2799954

[49] Knutson B, Wimmer GE, Kuhnen CM, Winkielman P (2008) Nucleus accumbens activation mediates the influence of reward cues on financial risk taking. Neuroreport 19: 509–513. 18388729 10.1097/WNR.0b013e3282f85c01

[50] Knutson B, Adams CM, Fong GW, Hommer D (2001) Anticipation of increasing monetary reward selectively recruits nucleus accumbens. The Journal of neuroscience : the official journal of the Society for Neuroscience 21: RC159. 11459880 10.1523/JNEUROSCI.21-16-j0002.2001 PMC6763187

[51] Clithero JA, Reeck C, Carter RM, Smith DV, Huettel SA (2011) Nucleus accumbens mediates relative motivation for rewards in the absence of choice. Frontiers in human neuroscience 5: 87. 21941472 10.3389/fnhum.2011.00087 PMC3171065

[52] Salamone JD (1994) The involvement of nucleus accumbens dopamine in appetitive and aversive motivation. Behavioural brain research 61: 117–133. 8037860 10.1016/0166-4328(94)90153-8

[53] Schlund MW, Cataldo MF (2010) Amygdala involvement in human avoidance, escape and approach behavior. NeuroImage 53: 769–776. 20600966 10.1016/j.neuroimage.2010.06.058 PMC2930061

[54] Ehrlich I, Humeau Y, Grenier F, Ciocchi S, Herry C, et al (2009) Amygdala inhibitory circuits and the control of fear memory. Neuron 62: 757–771. 19555645 10.1016/j.neuron.2009.05.026

[55] Debiec J, Diaz-Mataix L, Bush DE, Doyere V, Ledoux JE (2010) The amygdala encodes specific sensory features of an aversive reinforcer. Nature neuroscience 13: 536–537. 20348916 10.1038/nn.2520 PMC2860669

[56] Kahn I, Yeshurun Y, Rotshtein P, Fried I, Ben-Bashat D, et al (2002) The role of the amygdala in signaling prospective outcome of choice. Neuron 33: 983–994. 11906703 10.1016/s0896-6273(02)00626-8

[57] Barad M, Gean PW, Lutz B (2006) The role of the amygdala in the extinction of conditioned fear. Biological psychiatry 60: 322–328. 16919522 10.1016/j.biopsych.2006.05.029

[58] Bolla KI, Eldreth DA, Matochik JA, Cadet JL (2005) Neural substrates of faulty decision-making in abstinent marijuana users. NeuroImage 26: 480–492. 15907305 10.1016/j.neuroimage.2005.02.012

[59] Vaidya JG, Block RI, O'Leary DS, Ponto LB, Ghoneim MM, et al (2012) Effects of chronic marijuana use on brain activity during monetary decision-making. Neuropsychopharmacology : official publication of the American College of Neuropsychopharmacology 37: 618–629. 21956445 10.1038/npp.2011.227 PMC3260974

[60] Cousijn J, Wiers RW, Ridderinkhof KR, van den Brink W, Veltman DJ, et al.. (2012) Individual differences in decision making and reward processing predict changes in cannabis use: a prospective functional magnetic resonance imaging study. Addiction biology. 10.1111/j.1369-1600.2012.00498.x 22994937

[61] Cousijn J, Goudriaan AE, Ridderinkhof KR, van den Brink W, Veltman DJ, et al (2012) Approach-bias predicts development of cannabis problem severity in heavy cannabis users: results from a prospective FMRI study. PloS one 7: e42394. 22957019 10.1371/journal.pone.0042394 PMC3434213

[62] Bechara A (2005) Decision making, impulse control and loss of willpower to resist drugs: A neurocognitive perspective. Nature Neuroscience 8: 1458–1463. 16251988 10.1038/nn1584

[63] Verdejo-Garcia A, Bechara A (2009) A somatic marker theory of addiction. Neuropharmacology 56 Suppl 148–62. 18722390 10.1016/j.neuropharm.2008.07.035 PMC2635337

[64] Tzourio-Mazoyer N, Landeau B, Papathanassiou D, Crivello F, Etard O, et al (2002) Automated anatomical labeling of activations in SPM using a macroscopic anatomical parcellation of the MNI MRI single-subject brain. Neuroimage 15: 273–289. 11771995 10.1006/nimg.2001.0978

