# Supplementary material for: Neural Effects of Positive and Negative Incentives during Marijuana Withdrawal
Source: PLoS One. 2013 May 15;8(5):e61470. doi: 10.1371/journal.pone.0061470 (PMC3655173; doi:10.1371/journal.pone.0061470)
Supplement: Material S1 — Effects of incentive magnitude. (DOCX) [file pone.0061470.s001.docx]

**Supplemental Materials for**

**Neural Effects of Positive and Negative Incentives during Cannabis Withdrawal**

Francesca M. Filbey, PhD, Joseph Dunlop, MS, BA, and Ursula S. Myers, BA,

**Effects of incentive magnitude.**

A partially repeated measures ANOVA for RT with type of incentive (GAIN, LOSS) and level of incentive ($5, $1, $0.20) repeated, found no effect of incentive magnitude on response time (*F* (2, 166)= 0.46, MSE=148.8 *p*=0.6322), but did find a significant higher order interaction between incentive magnitude, group, and type of incentive; in the MJ group at the $5 incentive level, RT was faster for GAIN trials (M= 207.3) than in LOSS trials (M = 221.5) (*F* (2, 166)= 15.84, MSE=178.1, *p*<0.001). The table below lists the peak regions of activation for the controls (CON) during the high magnitude trials (i.e., $5.00) (cluster-corrected *p*<.05, *z*=2.3).

| ***GAIN vs. NEUTRAL (cluster size = 40,759)*** | | | | | |
| --- | --- | --- | --- | --- | --- |
| **Z** | **x** | **y** | **z** | **Localization** | **BA** |
| 4.71 | -56 | -26 | 24 | L inferior parietal lobe | 40 |
| 4.52 | 10 | 12 | 0 | R lentiform nucleus | - |
| 4.45 | -56 | -28 | 16 | L superior temporal gyrus | 42 |
| 4.27 | 20 | -62 | 2 | R lingual gyrus | 19 |
| 4.15 | -12 | -2 | 2 | L lentiform nucleus | - |
| 4.04 | -18 | 14 | -4 | L putamen | - |
| ***LOSS vs NEUTRAL (cluster size = 16,257 voxels)*** | | | | | |
| **Z** | **x** | **y** | **z** | **Localization** | **BA** |
| 4.41 | 50 | -24 | 46 | R postcentral gyrus | 2 |
| 4.3 | 30 | -48 | 64 | R superior parietal lobe | 7 |
| 4.08 | 52 | -6 | 0 | R superior temporal gyrus | 22 |
| 4.04 | 34 | -40 | 60 | R postcentral gyrus | 5 |
| 3.95 | 66 | -26 | -4 | R middle temporal gyrus | 21 |
| 3.88 | 60 | 6 | -4 | R superior temporal gyrus | 22 |
